# Recurrent hemolytic uremic syndrome

**DOI:** 10.4103/0971-4065.43694

**Published:** 2008-07

**Authors:** G. Lakshminarayana, R. Rajesh, A. Jojo, G. Kurian, V. N. Unni

**Affiliations:** Department of Nephrology, Amrita Institute of Medical Sciences, Kochi, India; 1 Department of Pathology, Amrita Institute of Medical Sciences, Kochi, India

**Keywords:** Hypocomplementemia, hemolytic uremic syndrome, recurrent acute renal failure

## Abstract

Hemolytic uremic syndrome (HUS) is an uncommon cause of acute renal failure. Diarrhea-associated (D+) HUS, usually seen in children, is a common variety of HUS. HUS that is not associated with diarrhea (D−) is caused by a heterogeneous group of disorders. We report here a case of recurrent HUS (D−) in an adult female with hypocomplementemia.

## Introduction

Hemolytic uremic syndrome (HUS) is an entity comprising of a triad of nonimmune hemolytic anemia, low platelet count, and renal failure.^1^ Diarrhea-associated HUS (typical HUS) is the most common variety and has a good prognosis, whereas the D- type (atypical HUS) is associated with a poor outcome.^1^ Some cases of atypical HUS like familial HUS, drug induced and malignancy-associated types can have recurrent episodes. Estimation of the levels of serum complement components in the patients and their family members can be a very useful test in familial HUS cases.^2^–^4^

## Case Report

A 26 year-old primipara who had undergone a lower segment cesarean section (LSCS) in September 2006 at another center, presented to us with oliguria, worsening edema, nausea, and vomiting a week after delivery. Increasing pallor and edema were noticed one week after LSCS that had been done near term due to fetal distress. The initial evaluation at a local hospital showed anemia, thrombocytopenia, and renal failure.

She was referred here for further management. She had no history of blood loss, drug intake, wound infection and a recent history of diarrhea. She had no other systemic symptoms. On examination, she was found to be oriented, afebrile, edematous, pale, and her blood pressure was 160/100 mm Hg. The LSCS wound was clean; her systemic examination and gynecological evaluation were normal.

**Fig. 1:**
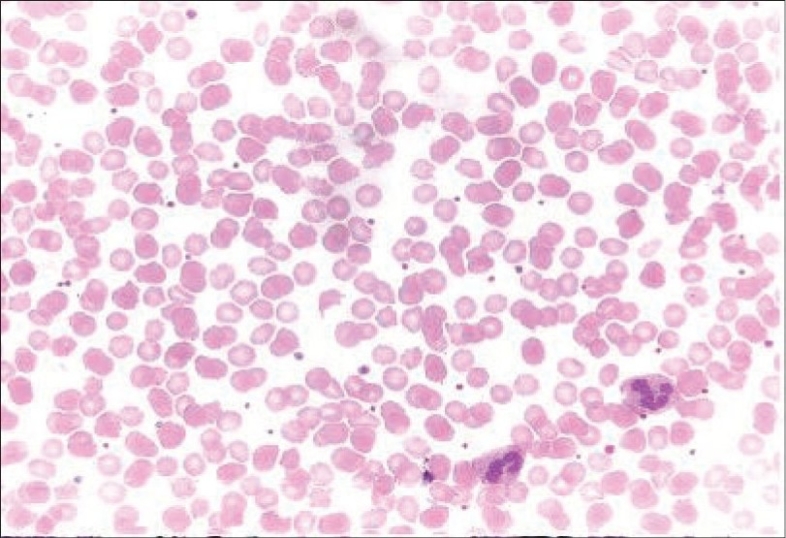
Peripheral blood smear showing schistocytes, helmet cells and burr cells

Investigations revealed Hb: 5.5 g/dL, platelets: 49,000/cu. mm, reticulocyte count: 10/dL, and a peripheral smear showed polychromasia, nucleated RBCs, helmet cells, schistocytes, and burr cells [Fig. 1] suggestive of microangiopathic hemolytic anemia. She had unconjugated hyperbilirubinemia with normal aspartate aminotransferase, alanine aminotransferase and alkaline phosphatase, and the lactate dehydrogenase (LDH) level was 7200 U/L. She was found to have severe renal failure (creatinine: 11.6 mg/dL) with 2+ proteinuria and microhematuria. PT/aPTT and fibrinogen were normal, blood and urine cultures were sterile; she had low serum C_3_ (Serum C_3_: 65 mg/dL, normal: 80–120 mg/dL) and normal C_4_ levels. Coomb's test was negative and antinuclear antibody as well as anti-ds DNA were negative. Bone marrow showed erythroid hyperplasia and; the ultrasound of the abdomen and pelvis was normal. The patient was diagnosed to have D – hemolytic uremic syndrome (D- HUS).

**Fig. 2:**
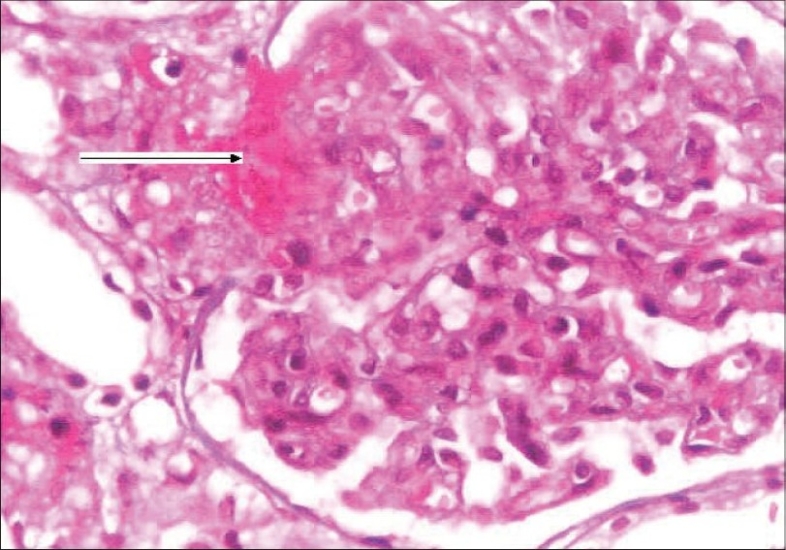
Kidney biopsy showing glomerulus with increase in mesangial matrix, focal endocapillary cell swelling and arterioles with platelet fibrin thrombi (arrow), and fibrinoid necrosis. (H&E stain, magnification ×400)

She was started on hemodialysis and after stabilization underwent a renal biopsy. Her renal biopsy [Fig. 2] showed glomeruli with an increase in the amount of mesangial matrix, focal endothelial cell swelling, wrinkling of the capillary walls, and fibrinoid necrosis. The arteries and arterioles showed platelet fibrin thrombi, fibrinoid necrosis of the vessel walls, fragmented RBCs, and nuclear debris. Immunoflouresence showed 2+ granular mesangial C_3_; C_3_ and fibrin were present in arterioles; IgG, IgA, IgM, and C1q were absent. These findings were consistent with a diagnosis of thrombotic microangiopathy.

The patient was treated with daily plasma exchange (plasmapheresis with plasma replacement of 40 mL/kg plasma was removed daily) for three weeks and alternate day hemodialysis. She remained anuric for two weeks which was followed by a gradual recovery of the platelet counts with an improvement in hemoglobin levels and urine output. She was discharged six weeks after admission with a Hb level of 10.5 g/dL, a platelet count of 240,000/cu. mm and a serum creatinine level of 2.4 mg/dL. She was advised regular follow-up as an outpatient. By end of three months she was asymptomatic, blood pressure remained under control with Nicardia retard 10 mg twice daily. Her Hb level was 11 g/dL, platelet count: 3 00,000/cu. mm, and serum creatinine: 1.2 mg/dL) and serum complement C_3_ level was 68 mg/dL.

**Fig. 3:**
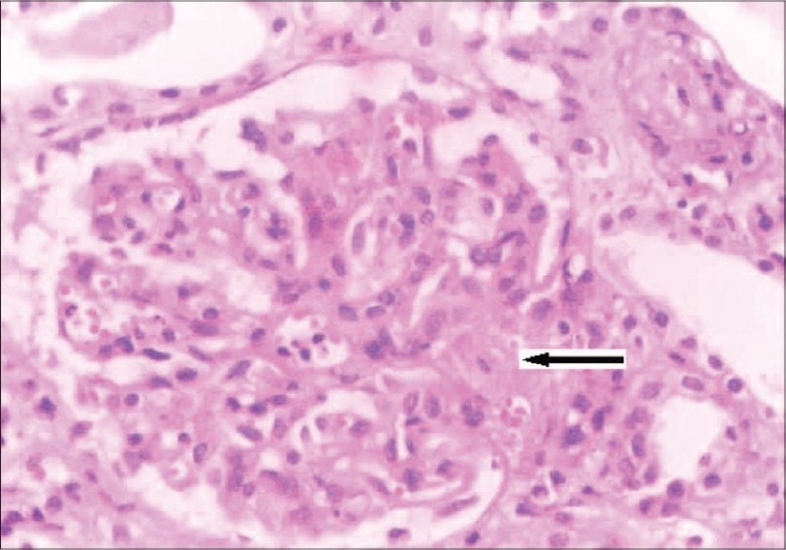
Kidney biopsy (on second admission) showing glomerulus with increase in mesangial matrix, focal endocapillary cell swelling and arterioles with platelet fibrin thrombi (arrow), and fibrinoid necrosis. (H&E stain, magnification ×400)

She was readmitted ten months later with uncontrolled hypertension, severe anemia, and renal failure. Evaluation showed severe renal failure (creatinine: 10.6 mg/dL), microangiopathic hemolytic anemia (Hb: 6.5 g/dL), thrombocytopenia (65,000/cu.mm) with an LDH level of 2850 U/L, serum complement C_3_ level: 60 mg/dL, and C_4_: 25 mg/dL. Kidney biopsy showed glomeruli with focal endocapillary cell swelling and fibrinoid necrosis. Some of the arteries and arterioles showed platelet fibrin thrombi and fibrinoid necrosis of the vessel walls. Immunoflouresence showed 2+ mesangial granular C_3_. The above findings were similar to those of her first biopsy, suggestive of thrombotic microangiopathy (Fig. 3). She was started on hemodialysis and daily plasmapheresis (40 ml/kg plasma was removed daily) for three weeks. She did not respond to therapy this time and she continues to be on hemodialysis even at this point of time. We evaluated her parents: her mother, who was asymptomatic, had hypocomplementemia (low C_3_: 70 mg/dL and normal C_4_) while the father had normal complement levels.

This 26 year-old female presented with recurrent severe renal failure due to thrombotic microangiopathy. She had low C_3_ levels and her mother had hypocomplementemia as well.

## Discussion

The diagnosis of HUS can be considered if a triad consisting of hemolytic anemia, thrombocytopenia, and renal failure occurs in a patient.^1^ The types of HUS are D+ or the typical type (Shiga toxin-associated), which forms the majority (90%) of all cases and the D– type or the atypical type that accounts for a minority (10%) of the cases.^1^ The latter type is caused by a heterogeneous group of disorders comprising of HIV and other viral infections, drugs (Calcineurin inhibitors, Ticlopidine, Clopidogrel, oral contraceptives), systemic lupus erythematosus, pregnancy / postpartum state, familial and posttransplant HUS.^1^

The D–type is usually seen in adults. It has a wide variety of precipitating factors as mentioned above, and its pathogenesis is poorly understood. It is idiopathic in about 50% of the cases. In general, the prognosis is poor with a mortality rate of 25% in the acute phase; common long-term sequelae in (50%) survivors are hypertension, chronic renal failure, and irreversible brain damage.^1^ Deficiencies of complement regulatory proteins such as factors H and I, as well as of MCP (membrane cofactor protein) have been found in some patients, and these can run in families. Normally factor H downregulates the activity of the alternate complement pathway. Factor H mutations are found in about 30% of patients with D- HUS.^2^ The MCP is a widely expressed transmembrane complement regulator that is abundantly found in the endothelium.^1^ Both factor H and MCP act as cofactors for factor I, which regulates complement activation by cleaving C3b.^1^

Familial HUS account for < 3% of all HUS cases. This entity is inherited as autosomal dominant (AD) or autosomal recessive; AD is more common in adults and has a poor prognosis.^1^ Low serum C_3_ and normal serum C_4_ levels are seen in patients. Low C_3_ levels have been found in healthy relatives of patients who have HUS.^1^,^3^ Genetic abnormalities of complement regulatory proteins (Factor H, Factor I, and MCP mutations) are seen in 50% of the patients.^3^ A significant association has been found between low C_3_ levels and factor H abnormalities.^1^–^4^ A low C_3_ level in a case of familial HUS is likely to be due to the inherited deficiency of factor H.^4^ *Factor H* gene is mapped on chromosome 1q. Factor H acts as a cofactor for the serine protease factor I, which degrades C3b.^4^ Deficiency of other complement regulatory proteins may also occur in familial HUS. These complement abnormalities make the individual susceptible to excessive complement activation by agents that are potentially toxic to the vascular endothelium such as certain viruses, bacterial toxins, immune complexes, and drugs.^4^ In familial HUS, a low C_3_ level in a family member is associated with a higher risk (sixteen times greater risk) of the disease.^4^

## Treatment of Atypical HUS

Plasma exchange is better than plasma infusion alone; one plasma volume (40 mL/kg) is exchanged per session and it should be started within 24 hours of presentation and continued for three weeks.^1^–^3^ Replacement with recombinant factor H (HF1) is an alternative treatment in FHUS associated with factor H mutations.^1^ Complement inhibitors (directed against C_5_) Pexelizumab and Eculizumab (humanized monoclonal antibodies) that are being tried in complement disorders may offer hope for future treatment.

Familial HUS has poor prognosis with a mortality rate of 25% in the acute phase. Those who survive have a high risk (50% in sporadic forms and 60% in familial forms) of progression to ESRD.^1^ Replacement therapy options for those who develop ESRD are maintenance dialysis (hemodialysis or peritoneal) and renal transplantation. Renal transplantation is associated with high rates of recurrence (50%) of HUS and graft loss and as there is no effective treatment of recurrences, graft failure occurs in >90% of them.^1^,^3^ Such patients should not receive another transplant as there is a very high chance of recurrence of HUS in the second transplant. Live-related donor transplant should also be avoided, as there is a risk for precipitation of HUS in the healthy relative, who may be predisposed to the disease. Many centers do not recommend live-related transplantation because of the risk for recurrence in the recipient and of de novo disease in the donor.^3^ The above mentioned poor outcome is for those with mutations or deficiency of factor H. Those with factor I and MCP deficiency-associated HUS have relatively better results after transplantation. Simultaneous kidney and liver transplant has been attempted as the liver is the source for synthesis of factors H and I.^1^–^3^

## Conclusion

HUS is one of the rare causes of acute renal failure, and has a poor prognosis. There is a need for early aggressive management in the form of prolonged plasmapheresis and hemodialysis support. Residual renal dysfunction is considered as the rule in 100% of the survivors. Our patient had a severe form of recurrent HUS associated with hypocomplementemia, which is very likely to be familial in nature.
